# Current Recommendations for Airway Management Techniques in COVID-19 Patients without Respiratory Failure Undergoing General Anaesthesia: A Nonsystematic Literature Review

**DOI:** 10.15388/Amed.2021.28.1.9

**Published:** 2021-02-19

**Authors:** Milda Grigonytė, Agnė Kraujelytė, Elija Januškevičiūtė, Giedrius Šėmys, Greta Bružytė-Narkienė, Oresta Kriukelytė, Eglė Kontrimavičiūtė, Nomeda Rima Valevičienė

**Affiliations:** Faculty of Medicine, Vilnius University, Vilnius, Lithuania; Faculty of Medicine, Vilnius University, Vilnius, Lithuania; Faculty of Medicine, Vilnius University, Vilnius, Lithuania; Faculty of Medicine, Vilnius University, Vilnius, Lithuania; Faculty of Medicine, Vilnius University, Vilnius, Lithuania Centre of Anaesthesiology, Intensive Therapy and Pain Management, Faculty of Medicine, Vilnius University, Vilnius, Lithuania; Faculty of Medicine, Vilnius University, Vilnius, Lithuania Centre of Anaesthesiology, Intensive Therapy and Pain Management, Faculty of Medicine, Vilnius University, Vilnius, Lithuania; Centre of Anaesthesiology, Intensive Therapy and Pain Management, Faculty of Medicine, Vilnius University, Vilnius, Lithuania; Department of Radiology, Nuclear Medicine and Medical Physics, Faculty of Medicine, Vilnius University, Vilnius, Lithuania

**Keywords:** anaesthesia, preoxygenation, airway management, difficult airway, SARS-CoV-2, COVID-19

## Abstract

**Summary. Background.:**

Since severe acute respiratory syndrome coronavirus 2 (SARS-CoV-2) first emerged, many articles have been published on airway management for coronavirus disease 2019 (COVID-19) patients. However, there is a lack of clear and concise conceptual framework for working with infected patients without respiratory failure undergoing general anaesthesia compared to noninfected patients. The aim of this article is to review current literature data on new challenges for anaesthesia providers, compare standard airway management techniques protocols with new data, and discuss optimisation potential.

**Materials and methods.:**

Literature search was performed in Google Scholar and PubMed databases using these keywords and their combinations: anaesthesia, preoxygenation, airway management, difficult airway, SARS-CoV-2, COVID-19. The following nonsystematic review is based on a comprehensive literature search of available data, wherein 41 articles were chosen for detailed analysis. Summarised and analysed data are presented in the article.

**Results.:**

SARS-CoV-2 has unique implications for airway management techniques in patients without respiratory failure undergoing general anesthesia. Main differences with the standard practice include: institutional preparedness, team composition principles, necessary skills, equipment, drugs, intubation and extubation strategies. Failed or difficult intubation is managed with predominance of emergency front of neck access (FONA) due to increased aerosol generation.

**Conclusions.:**

Airway management techniques in COVID-19 patients without respiratory failure are more challenging than in noninfected patients undergoing general anaesthesia. Safe, accurate and swift actions avoid unnecessary time delay ensuring the best care for patients, and reduce risk of contamination for staff. Appropriate airway strategy, communication, minimisation of time for aerosol generating procedures and ramped-up position aid to achieve these goals. During the pandemic, updated available literature data may change clinical practice as new evidence emerges.

## Abstract (lt)

**Dabartinės kvėpavimo takų valdymo technikų rekomendacijos COVID-19 pacientams be kvėpavimo nepakankamumo taikant bendrąją anesteziją: nesisteminė literatūros apžvalga:**

**Santrauka. Įvadas.**

Nuo sunkaus ūminio kvėpavimo sindromo koronaviruso 2 (SARS-CoV-2) atsiradimo buvo publikuota daug straipsnių apie COVID-19 sergančių pacientų kvėpavimo takų valdymą. Tačiau vis dar trūksta aiškios ir glaustos darbo su infekuotais pacientais be kvėpavimo nepakankamumo taikant bendrąją anesteziją, palyginti su neinfekuotais pacientais, koncepcijos. Šio straipsnio tikslas – apžvelgti dabartinius literatūros duomenis apie naujus iššūkius, su kuriais susiduria gydytojai anesteziologai, palyginti standartinių kvėpavimo takų valdymo technikų protokolus su naujais duomenimis ir aptarti darbo optimizavimo galimybes.

**Medžiaga ir metodai.**

Literatūros paieška buvo atlikta „Google Scholar“ ir „PubMed“ duomenų bazėse, vartojant šiuos raktinius žodžius ir jų derinius: *anestezija,* *preoksigenacija, kvėpavimo* *takų* *valdymas,* *sunkūs kvėpavimo* *takai,* *SARS-CoV-2,* *COVID-19*. Po peržiūros, pagrįstos prieinamų duomenų literatūros paieška, išsamiai analizei buvo pasirinktas 41 straipsnis. Apibendrinta ir išanalizuota medžiaga pateikiama šiame straipsnyje.

**Rezultatai.**

SARS-CoV-2 turi unikalią reikšmę parenkant kvėpavimo takų valdymo techniką pacientams be kvėpavimo nepakankamumo taikant bendrąją anesteziją. Pagrindiniai skirtumai, palyginti su standartine praktika, apima: bendrą pasirengimą, komandos sudėties principus, reikalingus įgūdžius, įrangą, vaistus, intubacijos ir ekstubacijos strategijas. Nepavykusi ar sunki intubacija yra valdoma atliekant skubią priekinę kaklo prieigą (FONA) dėl padidėjusios aerozolių generavimo rizikos.

**Išvados**

COVID-19 pacientų, kuriems nėra kvėpavimo nepakankamumo, kvėpavimo takų valdymo technikos yra sudėtingesnės, palyginti su neinfekuotais pacientais, kuriems taikoma bendroji anestezija. Saugūs, tikslūs ir greiti veiksmai leidžia išvengti nereikalingo delsimo, užtikrinama geriausia pacientų priežiūra ir mažėja darbuotojų užsikrėtimo rizika. Tinkama kvėpavimo takų valdymo strategija, bendravimas komandoje, aerozolius generuojančių procedūrų trukmės mažinimas ir taikoma pakeltos galvos padėtis padeda pasiekti šiuos tikslus. Pandemijos metu atnaujinti turimi literatūros duomenys gali keisti klinikinę praktiką atsiradus naujų mokslo įrodymų.

**Raktiniai** **žodžiai:** anestezija, preoksigenacija, kvėpavimo takų valdymas, sudėtingi kvėpavimo takai, SARS-CoV-2, COVID-19.

## Introduction

The number of coronavirus disease 2019 (COVID-19) cases are increasing worldwide due to human-to-human transmission of severe acute respiratory syndrome coronavirus 2 (SARS-CoV-2) [1].

The novel coronavirus is highly contagious therefore in just under a year many articles have been published on airway management strategies for infected patients due to high risk of viral transmission with reported incidence of infection being 1,07 % among surgical patients; in addition to this, transmission was reported to medical staff [2, 3]. Furthermore, this infection has a tendency of respiratory system involvement and progressive lung damage that results in even more challenging management of a patient in urgent surgical scenarios [4, 5]. These patients could be more susceptible to desaturation during apnea after anaesthetic induction due to pathophysiology of the COVID-19, therefore these patients require expert airway management techniques to ensure safety during anaesthesia [6]. However, there is a lack of clear and concise conceptual framework for anaesthesia providers working with SARS-CoV-2 positive patients without respiratory failure in comparison with noninfected patients. As the number of infected patients continues to mount, it becomes paramount to prepare operating theatre staff for the inevitable arrival of SARS-CoV-2 positive patients for urgent surgeries.

The aim of this article is to review current literature data on the new challenges anaesthesia providers face while working with SARS-CoV-2 infected patients without respiratory failure during aerosol-generating procedures of airway management with high risk of infection transmission, to compare the standard airway management and difficult airway protocols with new data in the literature during COVID-19 pandemic, and discuss optimisation potential ensuring the highest anti-infective safety standards while providing the best care for a patient.

## Materials and methods

Literature search was performed in Google Scholar and PubMed databases using these keywords and their combinations: anaesthesia, preoxygenation, airway management, difficult airway, SARS-CoV-2, COVID-19. The following nonsystematic review is based on a comprehensive literature search of available data, wherein 41 articles were chosen for detailed analysis. In the article, data for infected patients are summarised, analysed, and compared with those for noninfected patients.

## A review of literature

### Team

Preparation for airway management in SARS-CoV-2 positive patients brings new challenges to anaesthesia providers (Table 1).

**Table 1.** Preparation and plan before the procedure.

**PREPARE**			**PLAN**		
**Team**	**Personal protective equipment (PPE)**	**Equipment for endotracheal intubation**	**Strategy**	**Tracheal intubation checklist**	**Use techniques you have tried before and used for other patients**
An experienced doctor for intubation; a second doctor specialist; an assistant to give medication and monitor patient‘s vital signs.	Long sleeved gown; FFP3 mask; gloves; eyewear.	COVID-19 intubation trolley.	Preparation for routine intubation and for complicated intubation; all team members must be aware of procedure principles.	This list can help to reduce the risk of human errors during preparation for endotracheal intubation.	2-person 2-handed mask ventilation with a VE-grip; videolaryngoscopy is recommended.

Preparation for the procedure begins outside the operating theatre door: composition of the team consists of two doctors anaesthesiologists and an assistant, dressed in a full personal protective equipment (PPE) with double gloves, defog goggles and/or eyewear [7]. A checklist for routine and difficult intubation in SARS-CoV-2 infected patients should be presented and discussed; the checklist allows to follow an action plan if the intubation does not proceed as expected and further actions must be taken without confusion [2]. The team should choose the airway devices that they are most familiar with. Intubation should be performed by the most experienced airway manager [8, 9]. If the intubation fails on the first attempt, the other qualified physician can take over the procedure. A runner should be outside the operating theatre and be able to provide help rapidly if needed [10].

We take this new literature data into consideration and emphasise a need for specialised training for hospital staff to ensure smooth coordinated efforts and teamwork.

### Equipment

There are several differences in the equipment used in infected patients’ preoxygenation and endotracheal intubation in comparison with standard airway management. A separate COVID-19 intubation trolley should be prepared with the equipment required to intubate a patient safely, and brought into the operating theatre. The standard airway management trolley should also be kept outside the room; disposable single-use equipment should be used whenever possible [6]. The content of the COVID-19 trolley is similar to a standard difficult airway trolley, but there are some notable additions: at least 4 sets of PPE, a disposable Mapleson C circuit, and viral filters [7]. It is particularly important to attach a viral filter between the face mask and the anaesthetic circuit in order to avoid contamination of the circuit and contaminated gas expelling in the event of circuit disconnection [6]. Two filters (heat and moisture exchangers) should be fitted: one between the face mask and the elbow connector, and another at the expiratory limb of the anaesthetic circuit [2]. At least two laryngoscopes should be included in the set: a Macintosh direct laryngoscope and a Macintosh videolaryngoscope. As well as a hyperangulated videolaryngoscope should be included if available [6]. Due to fewer permissible failed intubations when performed by the most experienced airway manager in the team, videolaryngoscopy is a method of choice in COVID-19 patients [11]. Moreover, accurate, swift and safe intubation with videolaryngoscope helps to prevent episodes of desaturation, and doubles the distance between patient and operator faces [6, 12]. Wide range of endotracheal tubes (ETT), with preference of 7.0–7.5 mm ID for women and 8.0–9.0 mm ID for men, and the second generation supraglottic devices should be available [13, 14].

### Drugs

Several alternative sedative agents are used in COVID-19 patients. Ketamine is a drug of choice for induction of anaesthesia with a dose of 0.5–2 mg/kg administered intravenously; doses should be considered individually for patients with heart disease [15, 16]. In hypoxic and agitated patients with difficult uptake of oxygen during preoxygenation, it is advisable to reassure the patient with a lower dose of ketamine (0.5 mg/kg) than is normally given during rapid induction. This dose does not inhibit respiratory activity and the quality of spontaneous ventilation, patients are more tolerant of a sealed mask, thus increasing the amount of oxygen supplied and reducing the risk of spreading viruses in the air. When preoxygenation is complete, rapid induction is performed using the remaining dose of ketamine [17]. Bronchodilation, due to sympathomimetic effects of ketamine, can also benefit to improve lung function and reduce airway resistance. Due to safer effects on the heart compared to ketamine, etomidate can be another drug of choice; an intravenous dose ranges 0.1–0.3 mg/kg [18]. Etomidate affects respiratory function: coughing may occur, which increases the chance of the virus spreading. Due to rapid onset and haemodynamic profile, midazolam can be an alternative sedative agent; an intravenous dose is 0.02–0.03 mg/kg. At high doses, midazolam may reduce systemic vascular resistance. Propofol should be avoided in hypotensive patients [15].

The most commonly used opioid in COVID-19 patients during induction is fentanyl with a dose of 0.5 to 3 mcg/kg administered intravenously [19]. Alternative opioids (like remifentanil) could also be used due to a little effect on haemodynamics. However, at higher doses, opioids may inhibit the myocardium and hypotension may occur due to the release of histamine. In addition to this, histamine can cause bronchospasm and chest wall muscle rigidity, making ventilation and oxygen saturation difficult [15].

The choice of neuromuscular blocking agents for these patients remains unclear. Rocuronium (0.6–1.2 mg/kg), vecuronium (0.08–0.1 mg/kg) or succinylcholine (0.3–1.1 mg/kg) are recommended [20, 21]. Succinylcholine, due to release of histamine, can cause bronchoconstriction, and may have a greater effect on the cardiovascular system compared to rocuronium and vecuronium [15].

COVID-19 patients may require infusion of a vasopressor (e.g. norepinephrine), what is more, some patients at high risk of haemodynamic decompensation may require an intravenous pushdose pressor (e.g. phenylephrine 100–200 mcg) [17].

Salbutamol may be administered to minimise airway resistance in patients with reactive airway disease [22]. Available current literature does not provide the most optimal salbutamol form and route of administration. For sudden breathing difficulties we suggest using the patient’ personal salbutamol pressurised metered-dose inhaler (pMDI) before and after anesthesia, or starting perioperative intravenous salbutamol infusion in order to decrease the risk of viral spreading.

Drugs, such as remifentanil, lidocaine, and dexmedetomidine, reduce the risk of coughing and minimise agitation on extubation [23]. Administration of intravenous lidocaine prior to tracheal extubation can effectively reduce emergence coughing without any other significant side effects. Consideration should be given to injections of lidocaine at the beginning and the end of any procedure requiring intubation and/or extubation in patients with COVID-19 [24].

### Patient

#### Positioning

**Fig. 1.**
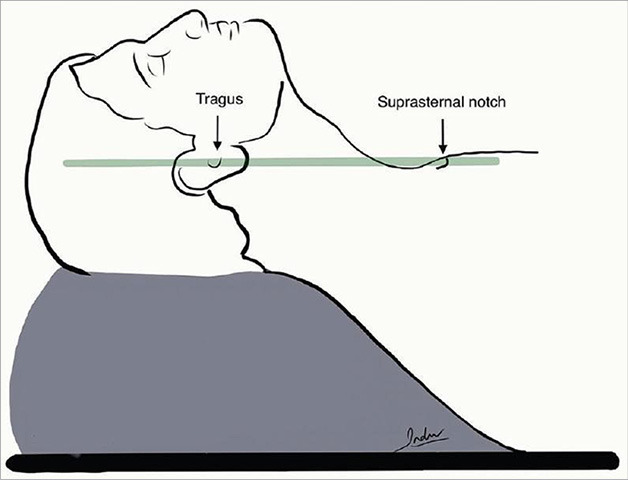
Ramped-up position.

Current literature data distinguish two main patient positions: 45° head up or ramped-up position (Fig. 1) [25, 26, 27, 28]. These positions are important for high risk patients (obese, hypoxaemic and critically ill) because they facilitate face mask ventilation, laryngoscopy and intubation, delaying the onset of hypoxia [25, 26].

#### Preoxygenation, face mask ventilation and endotracheal intubation

In noninfected patients spontaneous ventilation is maintained by supplying pure oxygen at a flow rate of 5 l/min for 2 to 5 min with the tight fitting face mask [29, 30]. The delay between the onset of apnoea and the occurrence of arterial oxygen desaturation (SpO2 90%) is limited to 1–2 minutes and can be extended to 6–8 min with pre-oxygenation in 100% inhaled oxygen [25, 29, 30]. Endtidal oxygen fraction 90% should be accomplished with eight deep breaths at an oxygen flow of 10 l/min within 1 min including noninvasive ventilation [25, 26, 27, 29, 30, 31, 32]. Nasal oxygenation is also useful in securing a tube when needed to extend the apnoea time in difficult intubation. Furthermore, apnoenic oxygenation, in addition to standard preoxygenation and face mask ventilation, is useful in high-risk patients and in healthy patients [25, 26]. Noninvasive positive pressure ventilation can be beneficial in hypoxic, obese or critically ill patients [31]. Intubation should be limited in maximum 2 attempts with direct laryngoscopy [29, 31, 32].

Due to aerosol-generating intervention, a completely sealed surgical mask and two layers of wet gauze covering the patient’s nose and mouth are also helpful. When endotracheal intubation is planned, two persons with 2-handed “vice grip” technique with 100 percent oxygen preoxygenation at a minimum of 5 min is mandatory [7, 10]. Two anaesthetic circuits have been advised: circle or a hand-held (eg. Mapleson C) with the minimum obligatory <= 6 l/min oxygen flow [2]. Similar to noninfected patients, a low-flow nasal oxygen therapy (flow rate <5 L/min) is helpful in patients at risk of hypoxia, in an attempt to extend the apnoea time [10, 29, 33]. High-flow nasal oxygenation is beneficial for a shorter intubation time and decreasing incidence of desaturation when compared with preoxygenation by face mask ventilation [33, 34]. Nevertheless, due to the high risk of aerosolization and virus transmission, we suggest to limit use of noninvasive ventilation and high-flow nasal oxygenation only in negative pressure rooms. The cuff should be inflated with air to a measured cuff pressure of 20–30 cm H2O in order to avoid cuff leak; if using high airway pressures, secure cuff pressure ≥ 5 cm H2O above peak inspiratory pressure and start ventilation only after cuff inflation [28]. Auscultation to ensure correct intubation may be inefficient due to wearing PPE, capnography curve appearance and return of Et carbon dioxide over several respiratory cycles is the golden standard. A second generation supraglottic airway with a preference for the second generation laryngeal mask airway that allows inserting an ETT assisted by fiberoptic bronchoscopy is a method of choice in the event of failed endotracheal intubation; though these devices do not completely seal the airway [13, 14].

#### Endotracheal extubation

The process of extubation in COVID-19 patients has several differences compared to the standard extubation sequence, and is designed to ensure the safety of operating theatre staff (Table 2) [35]. Only those directly involved should be present at the time of extubation [7]. Pre-extubation suction of oral secretions is important and a suction tube should be placed inside the patient’s mouth while removing the ETT [36]. Just before extubation an anaesthesia mask fitted with a plastic sheet draped over it is placed above the ETT and once the ETT is removed, the mask is sealed tightly over the patient’s mouth and nose [23, 36]. The plastic sheet serves as a physical barrier for droplets generated during extubation and it is removed once the patient is breathing and coughing has subsided [36]. Mechanical ventilation is stopped prior to extubation. Once a firm seal is established, the mask should be connected to a viral filter and then back to the anaesthetic circuit. When the face mask is no longer required, a nasal cannula should be inserted and a surgical face mask placed over the patient’s nose and mouth [23]. Coughing during extubation generates a significant amount of droplets and poses a risk of viral transmission to staff, therefore it’s advised to suppress the cough reflex by administering medications [37]. The use of protective barrier hood devices is an option, however, there is currently a lack of evidence for or against their use in extubation [38].

**Table 2.** Comparison of noninfected and COVID-19 infected patient airway management.

	**Noninfected patient**	**New for working with SARS-CoV-2 infected patient**
Position	Classic ‘sniffing’ 45° head up or ramped-up Reverse Trendelenburg or sitting	45° head up or ramped-up
Preoxygenation	Desaturation (SpO2 90%) limited to 1–2 min, can be extended to 6–8 min with pre-oxygenation in 100% inhaled oxygen Spontaneous ventilation 2–5 min, 5 L/min End-tidal oxygen fraction 90% with eight deep breaths within 60 seconds, 10 L/ min Face mask ventilation for 3 min High-risk patients – oxygen by nasal cannulae Nasal oxygen 15 L/min, or high flow nasal oxygen at 70 L/min	Airway assessment without removing the patient’s surgical mask Cover the patient’s nose and mouth with two layers of wet gauze 5 min (3–5 min) preoxygenation Hand-held circuit such as the Mapleson C, <= 6l/min O2 Face mask application with a 2-handed “vice grip” technique Tight fitting mask <5 L/min in patients at risk of hypoxia Use fiberoptic tracheal intubation High-flow nasal oxygenation is during rapidsequence induction and intubation
Face mask ventilation	Avoid if high risk of aspiration; Soon after induction and also between attempts at tracheal intubation; Face mask ventilation < 20 cm H2O; Mask ventilation with 100% oxygen	Avoid as aerosol generating procedure
Endotracheal intubation	Maximum 2 attempts Direct laryngoscopy	Most experienced/skilled airway manager Minimize attempts Rapid sequence induction Video laryngoscopy is recommended Auscultation may be ineffective if wearing PPE Inflate the cuff with air to a measured cuff pressure of 20–30 cm H2O If using high airway pressures, ensure cuff pressure ≥ 5 cm H2O above peak inspiratory pressure
Extubation	Assess for possible high risk extubation Ensure adequate preoxygenation with 100% O2 Perform airway suction before extubation Insert a bite block Antagonise neuromuscular blockade Awake extubation generally safer and preferred Deliver supplemental oxygen with nasal cannula in the recovery room In high risk scenarios consider deep extubation, laryngeal mask exchange, remifentanil infusion, airway exchange catheters	Place a suction tube inside the mouth Position a sealed anaesthesia mask with a barrier plastic drape over the ETT A viral filter should be attached to the anaesthesia mask Extubate while maintaining face mask seal and connect mask to anaesthetic circuit Switch to nasal cannula and place a surgical mask over patient’s mouth and nose once anaesthesia mask no longer required Minimal staff members should be present Pharmacologic suppression of cough reflex includes dexmedetomidine, lidocaine and opioids Use of barrier hood devices possible but lacking current evidence for or against

### Difficult airway management

Difficult airway management in noninfected patients in comparison with COVID-19 patients is based on the algorithmic approach according to difficult airway management.

There are some principles that are the same in working with both groups of patients. The initial tracheal intubation plan A is to adequately pre-oxygenate, perform a neuromuscular blockade, if necessary, do external laryngeal manipulation, intubate the patient, and further maintain oxygenation and anesthesia. If plan A is successful, an anesthesiologist must confirm tracheal intubation with capnography. In the event of failed intubation, the team must execute plan B. At plan B, a 2nd generation supraglottic airway device must be used with maximum 3 attempts. If plan B is failed, at plan C there is a possibility to use a face mask, including two person technique and adjuncts. The plan D is recommended to execute when the patient cannot be intubated and oxygenated. In the emergent front of neck access case, it is important to ensure neuromuscular blockade, position the patient in order to extend neck and perform a procedure [7, 39]. Comparison of differences in techniques for noninfected and COVID-19 patients’ difficult airway management is presented in Table 3.

### Cricothyroidotomy

Failed or difficult intubation is managed according to standard airway rescue algorithms with predominance of emergency front of neck access (FONA) due to risk of increased aerosol generation. Cricothyroidotomy must be performed as soon as possible when the patient cannot be intubated and ventilated. Firstly, it is important to exclude oxygen failure and blocked circuit and to ensure that this is maintained throughout airway management. Surgical cricothyrotomy should be the firstline procedure for ensuring airway function in COVID-19 patients (Table 3, plan D) [7]. There are various techniques and the choice must be made by the anaesthesiologist performing the procedure according to his experience and the preferable method, but the scalpel-bougie-tube technique is preferred in COVID-19 patients due to the risk of aerosolization with the oxygen insufflation associated techniques. The needle method is not recommended due to a small caliber of the needle, and therefore this method does not provide adequate oxygenation and ventilation [40, 41]. On the other hand cricothyroidotomy is rarely performed by anaesthesiologists, resulting in a lack of practical skills, thus the most experienced airway manager should perform this procedure if necessary.

## Discussion and conclusions

Adequate management of preoxygenation, endotracheal intubation and tracheal extubation in SARS-CoV-2 infected patients without respiratory failure for urgent surgery under general anaesthesia is more challenging than in noninfected patients. Safe, accurate and swift actions avoid unnecessary time delay for urgent surgery, ensure the best care for patients, and reduce risk of contamination for healthcare workers. Appropriate airway strategy, staff communication, minimisation of time for aerosol generating procedures and ramped-up position aid to achieve these goals.

There are some weaknesses in this nonsystematic review. Due to newly developed extremely contagious aerosol-generating manipulations for medical staff during preoxygenation, tracheal intubation and extubation episodes, a small number of recommendations (mostly institutional) limits the available data for review on this topic. Scientific literature data based on early evidence (less than 1 year of COVID-19 pandemic) and an absence of randomised controlled trials limit the possibility to choose a systematic review type of article. In this nonsystematic review article we focus on aerosol-generating procedures (preoxygenation, intubation of trachea and extubation, difficult tracheal intubation) techniques in patients without respiratory failure and compare these techniques with recommendations and routine practice in noninfected individuals. We do not discuss COVID‐19 patient’s mechanical ventilation strategies but rather we focus on airway management techniques. Challenges could be faced while ventilating COVID-19 patients during urgent surgery, especially in those with COVID-19 pneumonia, acute respiratory distress syndrome, and changed respiratory system mechanics resulting in impaired oxygenation and ventilation. However, patients with respiratory failure deserve attention and it will be the focus of the future article. We also do not discuss particularities managing morbidly obese, pregnant, eldery and pediatric patients.

**Table 3.** Comparison of non-infected and COVID-19 patients difficult airway management.

	Plan A: Face mask ventilation and tracheal intubation	Plan B: Maintaining oxygenation: SAD*** insertion	Plan C: Face mask ventilation	Plan D: Emergency front of neck access	Post-FONA care and follow up
**Noninfected patient**	Optimise head and neck position Pre-oxygenate Adequate neuromuscular blockade Direct/Video Laryngoscopy (maximum 3+1 attempts) External laryngeal manipulation Bougie Remove cricoid pressure Maintain oxygenation and anaesthesia	2^nd^ generation device recommended Change device or size (maximum 3 attempts) Oxygenate and ventilate	If face mask ventilation possible, paralyse Final attempt at face mask ventilation Use 2 person technique and adjuncts	Continue to give oxygen via upper airway Ensure neuromuscular blockade Position patient to extend neck Cricothyroidotomy	Postpone surgery unless immediately life threatening Urgent surgical review of cricothyroidotomy site Document and follow up as in main flow chart
	**Succeed:** confirm tracheal intubation with capnography **Declare failed intubation:** execute the plan B	**Succeed:** STOP AND THINK Options (consider risks and benefits): Wake the patient up Intubate trachea via the SAD Proceed without intubating the trachea Tracheostomy or cricothyroidotomy **Declare failed SAD ventilation:** execute the plan C	**Succeed:** wake the patient up Declare CICO****: CALL FOR HELP and execute the plan D		
**New for working with COVID-19 infected patient**	Staff must use full checked PPE* and share plan for failure Most appropriate airway manager to manage airway Position: head up if possible Pre-oxygenate: Mapleson C/Anaesthetic circuit – with HME Laryngoscopy (maximum 3 attempts) +/- bougie or stylet Maintain oxygenation (may use low flow, low pressure 2-person mask ventilation)	Plan B/C: Rescue oxygenation Maximum 3 attempts Change device/size/operator Open front of neck Airway set		Exclude oxygen failure and blocked circuit New staff must use full checked PPE Most appropriate airway manager to perform FONA	Closed tracheal suction Recruitment manoeuvre (if hemodynamically stable) Chest X-ray Monitor for complications Agree airway plan with senior clinicians
	**First failure:** Before entering room staff must don full checked PPE Get front of neck Airway (FONA**) set				

*PPE – personal protective equipment;

**FONA – emergency front of neck access in airway management;

*** SAD – supraglottic airway device;

****CICO – can’t intubate, can’t oxygenate.

During the pandemic, updated available literature data may change clinical practice as new evidence emerges.

## Conflict of interest

The authors declare that they have no conflict of interest.
